# Anti-NMDA receptor encephalitis and vaccination: A disproportionality analysis

**DOI:** 10.3389/fphar.2022.940780

**Published:** 2022-08-17

**Authors:** Salomé Martin, Brahim Azzouz, Aurore Morel, Thierry Trenque

**Affiliations:** Centre Régional de Pharmacovigilance et Pharmacoépidémiologie, Centre Hospitalier Universitaire de Reims, Reims, France

**Keywords:** anti-NMDA receptor encephalitis, vaccine, pharmacovigilance, drug safety, case-non case study

## Abstract

Anti–N-methyl-D-aspartate receptor (NMDAR) encephalitis is an auto-immune neurological disorder characterized by the presence in the cerebrospinal fluid (CSF) of antibodies against the GluN1 subunit of NMDA receptors in the brain. The etiology of the disease remains largely unknown. In this study, we aimed to investigate the possible existence of pharmacovigilance signals relating to a link between vaccination and the occurrence of anti-NMDAR encephalitis. We performed a case/non-case study using data from the World Health Organization pharmacovigilance database (VigiBase) up to 31 December 2021. All individual case study reports (ICSRs) linked to a vaccine and coded with the MedDRA Lower Level Term (LLT) “anti-NMDA receptor encephalitis” were analysed. We calculated the Reporting Odds Ratio (ROR) and 95% Confidence Interval (CI) for each type of vaccine. A total of 29,758,737 ICSRs were registered in VigiBase, of which 70 were coded under the selected LLT, and 29/70 (41.4%) involved a vaccine. Of these cases, 53.8% involved children aged younger than 15 years. The median time to onset of anti-NMDAR encephalitis after vaccination was 4 days (range 0–730). The highest RORs were observed for the diphtheria/polio/tetanus/pertussis vaccine [54.72 (95% CI 26.2–114.3)], yellow fever vaccine [50.02 (95% CI 15.7–159)] and human papillomavirus vaccine [32.89 (15.8–68.7)]. All cases were coded as serious; 13 patients did not recover, or were left with permanent sequelae. Nine patients recovered without sequelae or are on the path to recovery, and one patient died. In summary, pharmacovigilance signals were observed for anti-NMDAR encephalitis and vaccination. Clinicians need to be aware of this potential risk, and encourage to report any case of anti-NMDAR encephalitis occurring after vaccination.

## Introduction

Anti–N-methyl-D-aspartate receptor (NMDAR) encephalitis is an auto-immune neurological disorder first identified in 2007 (Dalmau et al., 2019), and characterised by the presence in the cerebrospinal fluid (CSF) of antibodies against the GluN1 subunit of the NMDA receptors in the brain (Florance et al., 2009; Irani et al., 2010; Dalmau et al., 2019). Incidence is estimated at around 1.5 per million persons annually (Dalmau et al., 2019). Anti-NMDAR encephalitis is more frequently observed in women (who account for around 80% of cases) and younger subjects, with a median age at diagnosis estimated at 20–22 years (Irani et al., 2010; Dalmau et al., 2019; de Bruijn et al., 2019). Patients with anti-NMDAR encephalitis generally present with convulsions, and abnormal movements and behaviours (Florance et al., 2009; Dalmau et al., 2019; de Bruijn et al., 2019). The presence of anti-NMDAR antibodies in the CSF confirms the diagnosis (Dalmau et al., 2019). Although the etiology of this disease is largely unknown, it often co-occurs with tumour (in around 20% of cases), notably ovarian teratoma, or with herpes simplex virus encephalitis (Irani et al., 2010; Dalmau et al., 2019; de Bruijn et al., 2019).

The widespread use of vaccines since their discovery has saved millions of lives from vaccine-preventable infectious diseases, and even enabled the eradication of diseases such as small pox. Vaccines work by educating the body’s immune system to recognize an infectious pathogen, thereby reducing substantially the risk of complications and mortality during later exposure to the pathogen (Canouï and Launay, 2019).

Although no link is currently known to exist between vaccination and the occurrence of anti-NMDAR encephalitis, a few rare cases have been reported in which a vaccine was the suspected trigger (Wang, 2017).

The aim of this study was to investigate the existence of potential pharmacovigilance signals between the administration of a vaccine and the occurrence of anti-NMDAR encephalitis, using data from the World Health Organization pharmacovigilance database.

## Materials and methods

### Case/non-study design

We performed a case/non-case study using disproportionality analysis, to evaluate the association between an adverse drug reaction (ADR) of interest and a drug. All reports of the ADR of interest are considered as cases, and all other reports are considered as non-cases. A two-by-two contingency table is used to calculate the Reporting Odds Ratio (ROR) which corresponds to ad/bc, where a = exposed cases, b = exposed non-cases, c = unexposed cases, d = unexposed non-cases. A signal deemed to exist when statistically significant disproportionality is demonstrated between cases and non-cases, namely:

➢ If the ROR = 1: No signal exists; the ADR of interest is as common with the drug of interest as with other drugs.
➢ If the ROR <1: No signal exists; the ADR of interest is less frequent with the drug of interest than with other drugs;
➢ If the ROR >1: The ADR of interest is more frequent with the drug of interest than with other drugs; there is thus a pharmacovigilance signal, and the higher the ROR, the greater the disproportionality (Montastruc et al., 2011; Faillie, 2019).

### Data source

VigiBase is the World Health Organization (WHO) global Individual Case Safety Report (ICSR) database. VigiBase has been collecting pharmacovigilance data since 1978, and now includes more than 30 million reports of ICSRs sent to the WHO Uppsala Monitoring Centre by the national pharmacovigilance systems of more than 150 countries. ICSRs are anonymized and coded according to the Medical Dictionary for Regulatory Activities (MedDRA^®^) (Brown et al., 1999). Each ICSR includes the patient’s demographic characteristics, the drug(s), the notifier, the characteristics of the ADR and the reporting country.

### Selection of cases and non-cases

We searched VigiBase up to 31 December 2021 to identify all ICSRs coded using the MedDRA Lower Level Term (LLT) “Anti-NMDA receptor encephalitis.” Each case was analysed to extract those where the suspected trigger was a vaccine. “Cases” corresponded to all ICSRs reporting anti-NMDAR encephalitis and suspected to be linked to a vaccine. Descriptive analysis of these cases is provided (age and sex of the patient, country, time to onset, severity, outcome).

“Non-cases” were all ICSRs recorded in VigiBase during the same time period, and not coded with the LLT.

### Statistical analysis

Using the data obtained from VigiBase, we calculated the ROR with 95% confidence intervals (CI) for each type of vaccine, classed according to the coded active ingredient. Vaccines with fewer than three ICSRs were excluded from statistical analysis. A *p*-value < 0.05 was considered statistically significant. Analyses were performed using SAS version 9.4 (SAS Institute Inc., Cary, NC, United States).

## Results

As of 31 December 2021, a total of 29,758,737 ICSRs were recorded in VigiBase, of which 70 were coded with the LLT “Anti-NMDA receptor encephalitis,” and 29 ICSRs of these were suspected to be linked to a vaccine. Overall, 56% of patients were women; median age was 13 years (range 1–69), and more than half (53.8%) were aged 15 years or younger, and a further 34.6% were aged 15–30 years.

In the 29 ICSRs, 51 vaccines were mentioned as suspect of involvement in the onset of anti-NMDAR encephalitis. The vaccines reported were the vaccines against: human papillomavirus (HPV), diphtheria/pertussis/tetanus/poliomyelitis (DTP-polio), influenza, varicella zoster, pneumococcal disease, Haemophilus influenzae type b (HIB), COVID-19, yellow fever, rabies, typhoid fever, hepatitis A/B, hepatitis A alone, and hepatitis B alone. The median time to onset after vaccination was 4 days (range 0–730).

Descriptive analysis of the cases shows that COVID-19, yellow fever, rabies, typhoid, hepatitis A/B vaccines were administered to adult patients (median age 25–36.5 years), contrary to the other vaccines, which were mainly administered to a paediatric population (median age 1.4–17.3 years).

All cases were reported to be serious (death = 1; life threatening = 3; caused/prolonged hospitalization = 16; disabling/incapacitating = 4; other medically important condition = 5). One third of reported cases came from Japan (34.5%), followed by France (17.2%) and the United Kingdom (17.2%).

Thirteen patients did not recover, or were left with permanent sequelae (44.8%); nine patients (31%) recovered or were recovering; one patient (3.5%) died. The outcome was unknown for six patients (20.7%).

The vaccines most commonly suspected of involvement in anti-NMDAR encephalitis were HPV (15.7%), DTP-polio (15.7%) and influenza (13.7%).

All four ICSRs involving the COVID-19 vaccine were messenger RNA vaccines.

The vaccines against rabies, hepatitis A and hepatitis B were excluded from further analysis due to the low (<3) number of ICSRs with each.

The highest RORs were observed with the DTP-polio, yellow fever and HPV vaccines (Table 1). Significant RORs were also observed for the vaccines against HIB, varicella zoster, influenza and pneumococcal disease. Only the COVID-19 vaccines were not associated with a significantly elevated ROR (Table 1).

**TABLE 1:** Reporting odds ratios for the risk of anti-NMDA receptor encephalitis in the WHO pharmacovigilance database VigiBase.

Drug (active ingredient)	“Anti-NMDA receptor encephalitis” cases with vaccine (n = 51)	Non “Anti-NMDA receptor encephalitis” cases with vaccine (n = 4,091,614)	ROR	95% CI	*p*-value
HPV Vaccine	8	116,283	32.89	[15.8–68.7]	<0.001
DTP-Polio vaccine	8	70,006	54.72	[26.2–114.3]	<0.001
Influenza vaccine	7	280,525	11.68	[5.3–25.5]	<0.001
Varicella zoster vaccine	5	193,141	11.78	[4.7–29.2]	<0.001
Pneumococcal vaccine	4	241,627	7.4	[2.7–20.3]	<0.001
HIB vaccine	4	89,544	20.08	[7.3–55.1]	<0.001
Covid-19 vaccine	4	2,881,218	0.57	[0.2–1.6]	0.262
Yellow fever vaccine	3	26,614	50.02	[15.7–159]	<0.001

## Discussion

In this study, we sought to determine whether administration of certain vaccines may be associated with an increased risk of the occurrence of anti-NMDAR encephalitis. We identified a pharmacovigilance signal for seven of the vaccine types investigated.

Overall, vaccines accounted for 28.5% of the drugs involved in ICSRs reporting anti-NMDAR encephalitis in VigiBase. The predominance of paediatric patients (53.8% aged <15 years) can be explained by the fact that vaccines are mainly administered in infants, children and adolescents, according to the WHO’s recommended routine immunization schedules for children. Furthermore, we noted that patient age differed according to the type of vaccine administered. The cases of anti-NMDAR encephalitis reported to have occurred after vaccination against COVID-19, yellow fever, rabies, typhoid fever and hepatitis A/B were observed principally among adults. This can be explained by the fact that adults may get these vaccines for the purposes of travel to endemic areas, or professional exposure (Haviari et al., 2015; Freedman and Chen, 2019; Pavli and Maltezou, 2022). In the worldwide vaccine strategy aimed at eradicating (or controlling) the current SARS-CoV-2 pandemic, children, adolescents and young adults were not prioritized to receive vaccines given the low rate of severe forms of disease among these groups, thus explaining the higher age among reported cases (Russell and Greenwood, 2021).

The ICSRs of anti-NMDAR encephalitis mainly came from countries with high rates of vaccine coverage, namely the European region (France and the United Kingdom), and the Western Pacific region (notably Japan) (Muhoza et al., 2021).

All the reported cases of anti-NMDAR encephalitis were serious, and 54.8% of patients did not recover, or were left with sequelae. In the literature, recovery or improvement is observed in around 80% of patients (Barbagallo et al., 2017; Wang, 2017; Dalmau et al., 2019). However, full recovery is slow, and sequelae of varying intensity may persist, including memory, humour or behavioural disorders (Irani et al., 2010; Barbagallo et al., 2017). Relapse has even been observed in 15%–25% of patients, albeit with less severe symptoms than during the first episode (Zeng et al., 2021).

According to our findings, a pharmacovigilance signal exists for the following vaccines: HPV, DTP-polio, influenza, varicella zoster, pneumococcal disease, HIB, and yellow fever.

Conversely, no signal was observed for the hepatitis A/B or COVID-19 vaccines.

Anti-NMDAR encephalitis is not mentioned in the Summary of Product Characteristics (SmPC) of any vaccine, despite the fact that several cases have been described in the literature after vaccine administration, notably vaccines against H1N1 influenza, DTP-polio, Japanese encephalitis or yellow fever (Dalmau et al., 2011; Hofmann et al., 2011; Wang, 2017; Guedes et al., 2021).

In 2010, Hofmann et al. (2011) reported the first case of anti-NMDAR encephalitis associated with vaccination in a 15 year old adolescent, who presented unusual fatigue and psychiatric symptoms in the weeks following administration of a TdaP-IPV booster. In 2011, Dalmau et al. (2011) reported the onset of anti-NMDAR encephalitis in two patients after vaccination against H1N1 influenza. Later, in 2017, Wang (2017) reported the case of a 2-year old infant who developed anti-NMDAR encephalitis 17 days after receiving a second dose of the vaccine against Japanese encephalitis. In parallel, Wang (2017) analysed the phylogenetic relationship of the microRNAs, which significantly regulate the vaccine viruses or bacteria, and the phylogenetic relationship of these viruses and bacteria. Their results showed that from a phylogenetic viewpoint, anti-NMDAR encephalitis could be caused by Japanese encephalitis vaccination, H1N1 vaccination or DTP-polio vaccination (Wang, 2017). More recently, three cases of auto-immune encephalitis after vaccination against yellow fever have been reported by Guedes et al. (2021), of which two were anti-NMDAR encephalitis; one case occurred in 14-year old girl and the second in a 39-year old woman. Anti-NMDAR antibodies were detected in the serum and the CSF, without any associated signs of cancer. A case report published in 2016 reported the case of a female 18 year old with low levels of anti-NMDAR antibodies in the serum following vaccination against HPV (Blitshteyn and Brook, 2017). However, no such antibodies were found in the CSF and the patient had no signs of encephalitis. We found no publications mentioning the occurrence of anti-NMDAR encephalitis after HPV vaccination, although the largest number of notifications in VigiBase implicates this vaccine.

Since the vaccines against COVID-19 are new, especially those using messenger RNA (mRNA) technology, there is currently no firm data available about their possible implication in the onset of auto-immune encephalitis. However, several publications have described cases of auto-immune encephalitis occurring after COVID-19 vaccination, regardless of the type of vaccine used. One such case involved a young women vaccinated with the Bnt162b2 vaccine (mRNA), who developed anti-NMDAR encephalitis with acute psychosis (Flannery et al., 2021). Another report described three patients who received the ChAdOx1 nCov-19 (non replicating viral vector) and who developed encephalitis (Zuhorn et al., 2021). A further publication reported the case of a 50-year old woman with a history of multiple sclerosis who presented a deterioration of neurological symptoms, with detection of anti-NMDAR antibodies in the serum. Two weeks previously, the patient had received the second dose of BBVIP-CorV vaccine (inactivated) (Etemadifar et al., 2022). Four cases of anti-NMDAR encephalitis were identified in VigiBase involving COVID-19 vaccines, all mRNA vaccines. This type of vaccine was the most widely used in the worldwide fight against SARS-CoV-2, as compared to the other vaccine types (WHO data - dedicated COVID-19 vaccination dashboard, available at https://covid19.who.int/). Therefore, their higher representation among the ICSRs is not unexpected. This hypothesis is strengthened by a descriptive study of the frequency of adverse events reported after COVID-19 vaccination, where the rate of ADRs reported was lower with mRNA vaccines than with live non-replicating vaccines (rate of adverse effects reported after the first injection: 92.2% with ChAdOx1 nCov-19; 82.0% with Ad26COV2.S vs*.* 81.7% with mRNA-1273 and 45.2% with Bnt162b2) (Kant et al., 2022).

The pathophysiological mechanisms mediating auto-immune encephalitis after vaccine administration remain to be elucidated, but several hypotheses have been proposed to explain this occurrence. One such hypothesis purports that there may be exacerbation of underlying systemic auto-immune disease further to activation of the immune response, provoking neuro-inflammation (Guedes et al., 2021; Zuhorn et al., 2021). Another theory is that certain microbial proteins induced by immunization with vaccines are similar to human proteins, and thus, the immune system could react to, and damage these self-proteins (Wang, 2017).

Despite the existence of a pharmacovigilance signal for certain vaccines, the benefits of vaccination should not be called into question, and remain largely superior to the risks.

To the best of our knowledge, this is the first study to investigate the existence of potential pharmacovigilance signals concerning vaccine administration and the potential occurrence of anti-NMDAR encephalitis. The wide use of the VigiBase reporting system in over 150 countries makes it possible to avail of universal and representative data from around the world. The case/non-case design is a validated method used in research and surveillance of drug safety (Pierfitte et al., 1999; Montastruc et al., 2011).

This study has some limitations. The main limit is that reporting is spontaneous, although serious ADRs are more often reported than mild ADRs. This type of encephalitis represents approximately 80% of all auto-immune encephalitis (Guan et al., 2016), and accordingly, of the 674 ICSRs recorded in VigiBase under the term “auto-immune encephalitis,” one might have expected a higher proportion to be anti-NMDAR encephalitis, and in any case, more than the 70 cases identified here. The search for anti-NMDAR antibodies in the serum and the CSF is not systematically performed in all countries, and therefore, there is likely under-diagnosis of anti-NMDAR encephalitis cases. Finally, the case/non-case method used in this study makes it possible to identify disproportionality in the reporting of cases of the event of interest with the drug of interest, but cannot be used to infer any causality between the drug and the event (Montastruc et al., 2011).

## Conclusion

This study confirms the potential existence of a link between vaccination and anti-NMDAR encephalitis. The etiology of this disease remains largely unknown, but clinicians need to be aware of this potential risk, and encouraged to report all cases of anti-NMDAR encephalitis occurring in patients who recently received a vaccine.

## Data Availability

The datasets presented in the current study are available with the permission of the UMC. Data are available from the corresponding author upon reasonable request.
